# Initiation of Liver Transplant in Nepal: A Milestone

**DOI:** 10.1155/2022/9461388

**Published:** 2022-10-14

**Authors:** Pukar Chandra Shrestha, Neeraj Joshi, Dipesh Lal Gurubacharya, Mohan Devbhandari, Aarati Rai, Tika Ram Bhandari, Prakriti Shrestha, Pragya Paneru, Subhash Gupta, Choon Hyuck David Kwon

**Affiliations:** ^1^Department of Transplant Surgery, Shahid Dharmabhakta National Transplant Centre (SDNTC), Bhaktapur, Nepal; ^2^Department of Gastroenterology and Hepatology, Shahid Dharmabhakta National Transplant Centre (SDNTC), Bhaktapur, Nepal; ^3^Department of Cardiothoracic and Vascular Surgery, Shahid Dharmabhakta National Transplant Centre (SDNTC), Bhaktapur, Nepal; ^4^Department of Anaesthesiology and Critical Care, Shahid Dharmabhakta National Transplant Centre (SDNTC), Bhaktapur, Nepal; ^5^Department of International Health, John Hopkins Bloomberg School of Public Health, Baltimore, USA; ^6^Department of Research, Shahid Dharmabhakta National Transplant Centre (SDNTC), Bhaktapur, Nepal; ^7^Department of Liver Transplant, Centre for Liver and Biliary Sciences, Max Superspeciality Hospital, Saket, Delhi, India; ^8^Department of Liver Transplant, Samsung Medical Centre, Seoul, Republic of Korea

## Abstract

**Background:**

The incidence of chronic liver disease is increasing in the Nepalese population. Liver transplantation (LT) is the best option for patients with end-stage liver disease (ESLD). Nepal's first liver transplant was performed in 2016 in an international collaborative effort at Shahid Dharmabhakta National Transplant Centre (SDNTC), Bhaktapur, Nepal. We aim to report details of the first five patients who had undergone liver transplantation in SDNTC before the beginning of the COVID-19 outbreak in the history of transplantation in Nepal.

**Method:**

A descriptive analysis of the clinical data of five adult recipients of liver transplantation at SDNTC was done. We described the patient's demographics, length of stay, and survival of all the first five patients who had undergone four living donor liver transplantations and one brain-dead donor liver transplantation in SDNTC before the beginning of the COVID-19 outbreak.

**Results:**

Recipients were between 36 and 63 years old. The recipients of the four live donor liver transplants (LDLT) and one brain-dead donor liver transplant (DDLT) had alcoholic liver disease and cryptogenic liver disease, leading to end-stage liver disease. The model for end-stage liver disease (MELD) scores ranged from 23 to 34. Out of five, four recipients and four donors are doing well and relishing the prospect of a normal life, while the recipient of a brain-dead donor liver transplant passed away due to postoperative primary graft failure.

**Conclusion:**

Despite the small number of liver transplants that have been done, the success of these has created confidence in a sustainable liver transplantation program in Nepal.

## 1. Introduction

Liver disease is a significant problem in Nepal [1]. Liver transplantation is the best treatment option for patients with end-stage liver diseases, acute liver failure, and selected cases of hepatocellular carcinoma [2]. The first successful liver transplant in the world was carried out by Thomas Starzl in 1967 [3]. It has been a milestone in the field of organ transplantation. Its applicability is expanding tremendously worldwide with better outcomes, and even developing nations have started performing it [4]. Liver transplants started in Nepal nearly 50 years after their inception in the medical world. The very first liver transplantation was conducted in December 2016 at the Shahid Dharmabhakta National Transplant Centre (SDNTC) situated in Bhaktapur, Nepal.

Introducing liver transplantation in a country like Nepal, where kidney transplantation was in its early stages and amidst the dearth of trained human resources and infrastructure, was a huge challenge. In addition, medical staff and the general population lacked awareness of the advantages of liver transplantation. Furthermore, misconceptions, false traditional beliefs, and fear about organ donation created problems in starting liver transplantation in Nepal. Nevertheless, with the opening of a dedicated transplant center and the persistent efforts of the transplant team, the dream became reality. It was only possible with the support from the South Korean and Indian transplant teams in supporting our transplant team to perform the transplant surgeries. Before the beginning of the COVID-19 outbreak, we had successfully performed the first five liver transplantations in SDNTC (4 LDLT and 1 DDLT). In this paper, we present the case scenarios of the patients and our experience with the first five liver transplants at SDNTC.

## 2. Methods/Results

A total of five patients underwent liver transplantation from four living donors and one brain-dead donor at the Shahid Dharmabhakta National Transplant Centre (SDNTC), Nepal, till the beginning of the COVID-19 outbreak included. All the recipients had end-stage liver disease for various reasons, including alcoholic liver disease and cryptogenic liver disease. All recipients were males, with ages ranging from 36 to 63 years. The demographic characteristics of patients and perioperative data are shown in Table 1. The consent for organ donation of brain-dead donor was provided by their relatives. All living donors had provided consent for liver donation for living donation LT. Ethical clearance was given by the transplantation coordination committee at SDNTC, Nepal.

**Figure 1:**
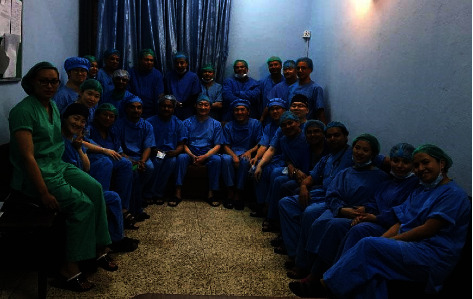
Group photo of the South Korean team and Nepalese team at SDNTC, Nepal, which was taken after the first liver transplantation.

**Figure 2:**
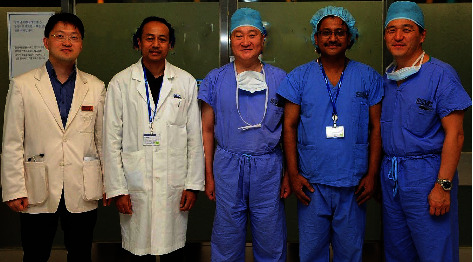
From right to left, Dr. Choon Hyuck David Kwon, Dr. Pukar Chandra Shrestha, Dr. Jae-won Joh, Dr. Neeraj Joshi, and Dr. Dong-Hyun Shin at the Samsung Medical Center, Seoul, South Korea, during the Nepalese team's exposure visit.

To set up a new liver transplantation program, the multidisciplinary team continuously worked hard to prepare themselves both technically by expanding the necessary technical skills as well as logistically. In our first case, which was an LDLT, we received tremendous support from the Korean transplant team, led by Dr. Jae-won Joh, Dr. Choon Hyuck David Kwon, and Dr. Gyu Seong Choi (Figures 1 and 2). While for the other three living donor liver transplantations, the Indian transplant team led by Dr. Subash Gupta from the Max Super Specialty Hospital supported us enormously. Meanwhile, one brain-dead donor liver transplant (DDLT) was performed entirely by the Nepalese team led by Dr. Pushkar Chandra Shrestha.

Regarding operative techniques, live donor hepatectomy was accomplished using a right subcostal incision with midline extension. In most cases, with the preservation of the left main branch, the right portal vein was divided. The middle hepatic vein, right hepatic vein, right hepatic artery, and right hepatic duct were separated and divided according to anatomical findings. The right hemiliver without MHV was taken out. The graft liver was perfused on the back table with an ice-cold HTK (Histidine Tryptophan-*α*-Ketoglutarate) solution.

The recipient's hepatectomy was done, and a temporary end-to-side portocaval shunt was created in two cases. In live donor liver transplantation, the MHV conduit and right hepatic vein were anastomosed to the inferior vena cava (IVC). The right portal vein was anastomosed to the main PV of the recipient. Grafting of the hepatic artery with the right hepatic artery and duct-to-duct anastomosis were done. In DDLT, IVC was anastomosed with IVC using the Piggy bag technique, and MPV was anastomosed with MPV. CHA was anastomosed with RHA and CHD was anastomosed with CHD (Table 1). An intraoperative doppler study was utilized to demonstrate vascular patency and satisfactory perfusion of the graft in each case.

Our first liver transplant recipient received induction immunosuppressive therapy with ATG and methylprednisolone. All others received methylprednisolone only at the time of surgery, followed by prednisolone, tacrolimus (a calcineurin inhibitor (CNI)), and mycophenolate mofetil as maintenance immunosuppression. The doses of these drugs were accustomed according to complete blood counts and based on serum tac levels and body weights.

All the postoperative outcomes were documented. The length of hospital stays ranged from 14 days to 16 days. The donors of four transplants had an almost uneventful recovery. All four recipients are leading normal lives with adequate immunosuppressive therapy. They have been followed up for a minimum of two years and a maximum of five years (Table 2).

Regarding postoperative events, our first live donor liver transplantation recipient developed hepatic artery thrombosis (HAT) on the third postoperative day, which was suspected by a Doppler scan and later confirmed by CT Angiogram. We thoroughly explored the artery and performed a thrombectomy redo the anastomosis of the artery, which resulted in the successful resumption of blood flow. The patient recovered well and was discharged on the 14^th^ POD.

A CT scan in the recipient of the third LDLT showed a pocket of purulent collection in the abdominal wall connected to the intraperitoneal. We re-explored the site on the 10^th^ POD and drained the pus. The pus culture report showed no growth and did not respond to antibiotics. Consequently, the patient was referred to the Max Super Specialty Hospital, Delhi under Dr. Gupta's team for further assessment. The team diagnosed him with aspergillosis, which is a rare complication in a liver transplant recipient with a high mortality rate. However, we were lucky to have this patient fully recovered following successful antifungal treatment.

Our fourth patient who underwent a brain-dead donor liver transplant developed postreperfusion diffuse bleeding, hypotension, and acidosis. However, there was no evidence of bleeding from the anastomosis site, so the abdomen was packed with three packs. The packs were removed on the second POD to check the bleeding status, but seeing no progress, the wound was packed again. The third look on day 3 showed no improvement either, except bleeding from raw dissection areas. The patient was on a cell saver to retransfuse the blood loss, which continued until the next day. With no success even after repeated attempts, the patient was pronounced dead on the 3^rd^ POD (i.e., 72 hours). We suspect primary graft failure and bleeding, possibly due to disseminated intravascular coagulation (DIC), as the possible causes of the event.

## 3. Discussion

Shahid Dharmabhakta National Transplant Centre (SDNTC) was established with the distinctive purpose of reinforcement and extension of organ donation and transplantation facilities in Nepal. The center executed its first live donor liver transplant in 2016 within a few years of establishment and played a pivotal role in the amendment of the Old Organ Transplant Act of 2000. Regarding liver transplantation, a multidisciplinary liver transplantation team was formed by the incorporation of concerned departments of the institute. The surgical proficiency of the team was improved by training in overseas liver transplantation centers. Academic discussions and work-ups were accompanied by the team leaders with government and nongovernment institutions and other concerned authorities. Leaflets and handouts on liver transplantation were circulated. Electronic and print media also helped in raising awareness of liver transplantation in the country.

Liver transplantation is still one of the most complicated techniques that requires a multidisciplinary approach. LDLT requires the highest level of expertise. Additionally, it is expensive and requires good perioperative care. The liver transplantation program was met with skepticism and suspicion in Nepal in the early days. Traditional and religious beliefs, absence of public awareness, dangers of commercialization, absence of a financial support system, and a small number of well-equipped centers and facilities for liver surgery were considered reasons for the failure of not having a program [5].

Meanwhile, after a decade of successful kidney transplantation in Nepal, the first successful liver transplant was performed in December 2016. It created a history in the field of transplantation in Nepal. However, the expansion of the liver transplantation program has been slower than expected. A total of four successful living donor liver transplants and one unsuccessful cadaveric liver transplant were performed over a three-year period at the SDNTC.

To date, there are a few government-approved centers, including SDNTC (government), TUTH (semigovernment), and a few teaching hospitals in Nepal to conduct liver transplantation. In all of these hospitals, liver transplants have been performed with the support of an experienced international liver transplant team following international practice. We have been fortunate to receive support from the South Korean and Indian teams to perform a number of liver transplantations at our center. However, the sustainability of the liver transplant program depends on various factors. One of the most vital ones is having a very devoted, properly trained, and experienced team. With the offering of organized training courses within the country, current limits in surgical and medical manpower are likely to be addressed along with the training program by international collaborations.

Organ transplantation is a costly procedure, and liver transplantation is one of the most expensive processes worldwide. Due to this, health service providers cannot provide services to even eligible patients for transplant unless that patient has the economic resources to pay for the transplant services. The costs are skyrocketed when the service is not available in the home country and people have to travel abroad for the service. In addition to this, the substantial cost related to the patient's postoperation care and antirejection medications increases a heavy annual expenditure on the existing economic load related to the procedure [6].

However, there are certain ways that can minimize the cost. Insurance should be one of the important ways to make the service easily accessible in the home country. In our context, due to numerous sociopolitical and financial reasons, insurance coverage has not gained extensive acceptance in Nepal. Moreover, the sustainability of insurance programs in developing countries is in trouble for various reasons, including information asymmetry, moral hazard, and lack of confidence in insurance policies [7]. Therefore, financial constraints continue to be the prime challenge in making liver transplants available to patients in need. Therefore, only if the government can implement a national policy to provide maximum financial support for liver transplant recipients or launch insurance policies targeted toward transplant services, can the number of people receiving the service increase. Likewise, the increased involvement of nonprofit organizations can minimize the burden to some extent.

Various articles around the world, especially from resource-limited settings, show that countries had to go through similar challenges to establish and sustain the liver transplantation service [8, 9]. Most transplant centers often struggle to regularize liver transplants due to various factors like financial limitations, political situations, the absence of inefficient organ procurement organizations, a lack of trained human resources, and infrastructural constraints [10].

An article from Taiwan, where the first LDLT was conducted in 1999, explains that the initial technical and infrastructural challenges were overcome by training the surgeons in the US and Japanese transplant centers despite language and diplomatic barriers. Nevertheless, coordinated animal experiments, organ donation legislation frameworks, expansion of hospital resources, laboratories, personnel training, development of perioperative radiologic imaging, transplant anesthesia, pathology and gastroenterology, refinement of donor hepatectomy procedure, and cooperation among Asian liver surgery centers eventually led to the successful establishment of liver transplant in Taiwan [11].

Likewise, Vietnam witnessed its first liver transplant in 2004 with the technical assistance of Belgium's transplant team. However, before this, for the preceding four years, Vietnam had been preparing its physical infrastructure and had been training its doctors in France and Belgium with short- and long-term courses on liver transplantation [12].

In Bangladesh, to overcome the challenges of setting up a new liver transplantation program, they formed a dedicated transplant center, department, and multidisciplinary team. The surgical team initially dissected cadaveric donors to get the basic concept of hepatic surgery and liver transplant. They even practiced upon the livers of animals, like cows, pigs, and sheep, to get more ideas about vascular and biliary anastomosis. Later, the transplant surgeons and other teams were further trained overseas. Thus, with the hard work of the multi-disciplinary team, in terms of infrastructure, technical expertise, well-equipped operation theatres, transplant ICU, and other resources, Bangladesh was all set to start liver transplantation in 2010 [13].

Pakistan performed its first liver transplant in 2003 with technical assistance from the international transplant team. However, the program was halted till 2012 due to various challenging factors like a lack of a skilled workforce, a mismatch in demand and supply of organs, a lack of awareness in the population, a dearth of outcome assessments, financial constraints, and most importantly, security and visa concerns of international teams. This dependence on external experts strengthened the need for developing local human resources, and finally, starting in 2012, various hospitals started accepting and mitigating such challenges with structured training programs for the transplant team. Pakistan has already conducted more than 500 liver transplants by February 2017 [14].

The first liver transplant in the Philippines was performed in 1988 on a brain-dead donor. Although it has established a sound liver transplant program, the main challenge at present is the shortage of grafts due to the low rate of deceased donor organ donation as seen in a majority of Asian countries. The graft shortage can be attributed to cultural and religious superstitions, lack of government funding, and lack of awareness of organ donation [15]. A similar challenge in liver transplantation has been reported in Malaysia [16].

Regarding the type of liver donation, the developed world performs more cadaveric donor transplants, while most live donor liver transplants (LDLTs) have been performed in developing countries [17]. To date, the main focus has been on living donor liver transplantation in our context. As brain death donation has been legalized in Nepal, there is a high possibility of developing cadaveric liver transplants in the future. Moreover, the amendment of the act in 2015 opened the doors for liver transplants and organ transplants from brain-dead donors [18]. Although many efforts are being executed to encourage deceased organ donation, the unique amalgam of demographic, economic, social, and political factors will most likely dominate deceased liver transplantation for a good number of years [19]. Even in the nations that have well-established liver transplant programs, it has been seen that they experience marked barriers to access, mostly contributed to factors like low deceased donation rates, lack of trained retrieval teams, scarce intensive care units, and inability to maintain the viability of donated organs [10].

Although only a small number of liver transplants have been performed in Nepal, the success of these has created hopes for a sustainable liver transplant program. Moreover, the transplant movement will continue to increase soon in Nepal due to the terrific problem of chronic liver diseases. The government will have to deliberate on inventive notions comprising private-public sector partnerships as well as proper incentivization of transplant recipients to make liver transplantation accessible to the needy in Nepal. The National Transplant Registry system should be transparent to endorse clearness in numerous phases of organ donation and outcome writing while providing transplant facilities with the highest standard of care.

## 4. Conclusions

Liver transplant is one of the most complicated procedures in medical science. Despite many challenges, liver transplantation has become possible in Nepal, and the performance of four successful liver transplants at SDNTC has so far generated a positive impact. This is also considered a landmark initiative for the continuation of liver transplantation services in Nepal. Although we have been relying on support from foreign experts at present, rapidly building newer health facilities with advanced infrastructure and increasing the number of trained human resources in this orbit should enable us to continue and expand this much-needed service in the most affordable and accessible way for the Nepalese population soon. While it would be unrealistic to expect rapid dramatic transformation, sustained commitment from the government, strategies to increase deceased organ donation, patience, optimism, commitment, and perseverance as learned from the experiences of other successful countries, and most of all, cooperation from the international transplant community will surely make this journey impactful.

## Figures and Tables

**Table 1:** Patient's demographics and perioperative data.

Variables	Patient 1	Patient 2	Patient 3	Patient 4	Patient 5
Recipient's age (years)	36	57	43	51	63
Recipient's sex	Male	Male	Male	Male	Male
Recipient's weight	62	65	50	54	53
Recipient's blood group	O positive	O positive	A positive	B positive	O positive
Recipient's medical illness leading to ESLD	Alcoholic liver disease	Alcoholic liver disease	Cryptogenic liver disease	Alcoholic liver disease	Nonalcohol steatohepatitis
Child-Turcotte-Pugh (CTP) score	B	B	B	C	B
Model for end-stage liver disease (MELD) score	23	24	24	26	34
Graft-to-recipient weight ratio (GRWR)	1.05	1.1	1.1	—	1.3
Donor age (years)	41	24	39	38	35
Donor sex	Female	Female	Female	Male	Male
Donor type	Live	Live	Live	Brain-dead	Live
Relationship to recipient	Sister	Daughter	Wife	Brain-dead donor	Son
Donor blood group	O positive	B positive	O positive	O negative	O positive
Cold ischemia time (mins)	85	22	82	660	84
Second warm ischemia time surgery (mins)	35	31	36	55	37
Venous anastomosis	d RHV to *r* RHV	d RIHV to *r* RHV	d RHV to *r* RHV Neo MHV to *r* MHV opening	d IVC to *r* IVC	d RHV to *r* RHV Neo MHV to *r* MHV opening
Portal anastomosis	d RPV to *r* RPV	d RAPS/RPPS to *r* MPV	d RPV to *r* MPV	d MPV to *r* MPV	d RPV to *r* MPV
Arterial anastomosis	d CHA to *r* RHA	d RHA to *r* LHA	d CHA to *r* RHA	d CHA to *r* RHA	d CHA to *r* LHA
Duct anastomosis	d RHD/LHD made single CHD to CBD	d RHD to CHD	RHD to CHD	d CHD to CHD	d RHD to CHD
Induction therapy	ATG and MP	—	—	—	—
Maintenance therapy	Tac, MM, steroid	Tac, MM, steroid	Tac, MM, steroid	Tac, MM, steroid	Tac, MM, steroid

d: donor; r: recipient; RHV: right hepatic vein; RIHV: right inferior hepatic vein; MHV: middle hepatic vein; RPV: right portal vein; MPV: main portal vein; RAPS: right anterior portal system; RPPS: right posterior portal system; CHA: common hepatic artery; RHA: right hepatic artery; LHA: left hepatic artery; RHD: right hepatic duct; LHD: left hepatic duct; CBD: common bile duct; ATG: antithymocyte globulin; MP: methylprednisolone; Tac: tacrolimus; MM: mycophenolate mofetil.

**Table 2:** Postoperative data.

Recipient	Patient 1	Patient 2	Patient 3	Patient 4	Patient 5
Re-exploration	Abdomen-thrombectomy for hepatic artery thrombosis	None	Wound site for collection	None	None
Rejection	No	No	No	Primary graft failure	No
Infectious complication	None	None	Aspergillosis	None	None
Vascular complication	None	None	None	None	None
Length of hospital stay (days)	14	16	12 and referred to India	Death on 3rd POD	16
Duration from operation	5 Years	4.5 Years	3.4 Years	3 Days	2 Years
Graft loss	None	None	None	Yes	None
Recipient's death	No	No	No	Yes	No

POD: postoperative day.

## Data Availability

The data used to support the findings of this study are available from the principal author and the corresponding author upon reasonable request.

## Abbreviations

LDLT: Live donor liver transplantation
SDNTC: Shahid Dharmabhakta National Transplant Centre
TUTH: Tribhuvan University Teaching Hospital
HAT: Hepatic artery thrombosis
CLD: Chronic liver disease
DIC: Disseminated intravascular coagulation
NASH CLD: Nonalcoholic steatohepatitis chronic liver disease

## Additional Points

Research registry no: 8357 (https://www.researchregistry.com/browse-the-registry#home/).
